# Three New Indole Diterpenoids from the Sea-Anemone-Derived Fungus *Penicillium* sp. AS-79

**DOI:** 10.3390/md15050137

**Published:** 2017-05-12

**Authors:** Xue-Yi Hu, Ling-Hong Meng, Xin Li, Sui-Qun Yang, Xiao-Ming Li, Bin-Gui Wang

**Affiliations:** 1 Laboratory of Marine Biology and Biotechnology, Qingdao National Laboratory for Marine Science and Technology, Key Laboratory of Experimental Marine Biology, Institute of Oceanology, Chinese Academy of Sciences, Nanhai Road 7, Qingdao 266071, China; huxueyi14@mails.ucas.ac.cn (X.-Y.H.); m8545303@163.com (L.-H.M.); lixin871014@163.com (X.L.); suiqunyang@163.com (S.-Q.Y.); lixmqdio@126.com (X.-M.L.); 2 College of Earth Science, University of Chinese Academy of Sciences, Yuquan Road 19A, Beijing 100049, China

**Keywords:** endophytic fungus, *Penicillium* sp., secondary metabolites, antimicrobial activity

## Abstract

Three new indolediterpenoids, namely, 22-hydroxylshearinine F (**1**), 6-hydroxylpaspalinine (**2**), and 7-*O*-acetylemindole SB (**3**), along with eight related known analogs (**4**–**11**), were isolated from the sea-anemone-derived fungus *Penicillium* sp. AS-79. The structures and relative configurations of these compounds were determined by a detailed interpretation of the spectroscopic data, and their absolute configurations were determined by ECD calculations (**1** and **2**) and single-crystal X-ray diffraction (**3**). Some of these compounds exhibited prominent activity against aquatic and human pathogenic microbes.

## 1. Introduction

**Figure 1:**
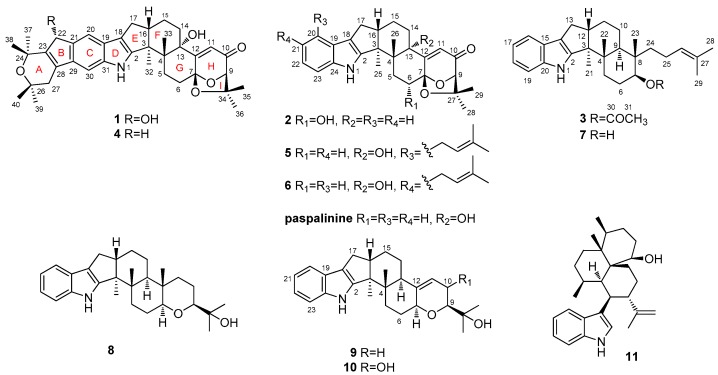
Structures of **1**–**11** and paspalinine.

Indolediterpenoids (IDTs) are a group of fungal metabolites that have attracted the attention of natural product chemists due to their diversified structures and potent biological activities [1,2]. IDTs usually possess a common core structure comprised of an indole moiety and is most likely derived from indole-3-glycerol phosphate and a geranyldiphosphate-derived cyclic diterpenoid skeleton [3]. The typical IDTs reported from fungal sources are penitrems, janthitrems, lotitrems, aflatrems, paxilline, and shearinines [2]. Previous reports revealed that IDTs possessed various bioactivities including antibiotic [4,5] and anti-insectan potency [6,7] as well as tremorgenic mammalian mycotoxicity [8,9]. During our investigation on marine-derived fungi, a series of structurally interesting secondary metabolites such as cyclohexadepsipeptides [10], diketopiperazine alkaloids [11], and diphenylketones [12] were discovered. Recently, we focused our attention on *Penicillium* sp. AS-79, a fungal strain isolated from the fresh tissue of the sea anemone *Haliplanella luciae*, and, as a result, three new (**1**–**3**) and eight known (**4**–**11**) indolediterpenoids are isolated and elucidated (Figure 1). Details of the structure determination and biological activities of these compounds are presented herein.

## 2. Results and Discussion

### 2.1. Structure Elucidation of the New Compounds

**Figure 2:**
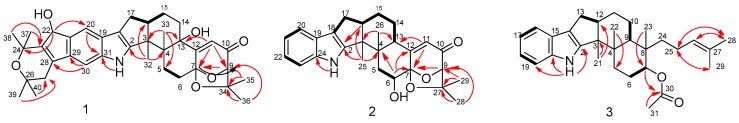
Key COSY (bold line) and HMBC (arrow line) correlations of Compounds **1**–**3**.

Compound **1** was obtained as a colorless solid. Its molecular formula was determined as C_37_H_45_NO_6_ on the basis of (+)-HRESIMS data, with 16 degrees of unsaturation. The ^1^H NMR data (Table 1) revealed the presence of eight methyl singlets and six methines (including three aromatic/olefinic and two oxygenated) as well as six methylenes in **1**. Its ^13^C NMR and DEPT data (Table 1) showed the presence of 37 carbon resonances, including eight methyls, six sp^3^ methylenes, six methines (including three aromatic/olefinic, two oxygenated and/or heteroatom-bonded, and one sp^3^ methine), and 17 quaternary carbons. The ^1^H and ^13^C data of **1** are very similar to those of shearinine F (**4**), an indolediterpenoid isolated from the marine mangrove-derived endophytic fungus *Penicillium* sp. (strain HKI0459) [13], indicating that **1** is also an indolediterpenoid derivative. Detailed comparison of the NMR data between **1** and **4** revealed that the methylene group at *δ*_C_ 36.2/*δ*_H_ 3.38 (CH_2_-22) of **4** was replaced by an oxygenated methine group at *δ*_C_ 76.3/*δ*_H_ 5.19 (CH-22) (Table 1). The observed HMBC correlations from H-22 to C-20 and C-24 confirmed the replacement of C-22 methylene in **4** by oxymethine in **1** (Figure 2).

**Figure 3:**
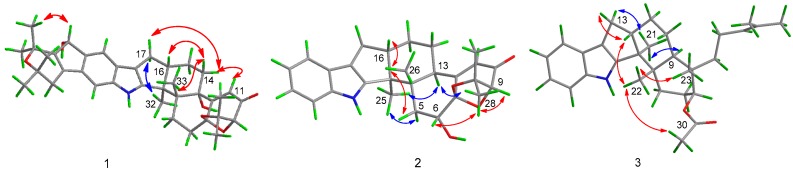
NOESY correlations of Compounds **1**–**3**.

**T**he relative configuration of **1** was determined by the NOESY spectrum. The NOESY correlations from H-32 to H-17*α*, from H-14*β* to H-16 and H-33, and from H-14*α* to H-11 confirmed the *trans* fusion of Rings E and F and of Rings F and G. For Rings A and B, the NOESY correlation between H-22 and H-38 placed the OH group on C-22 and CH_3_-38 on the opposite face (Figure 3).

**Figure 4:**
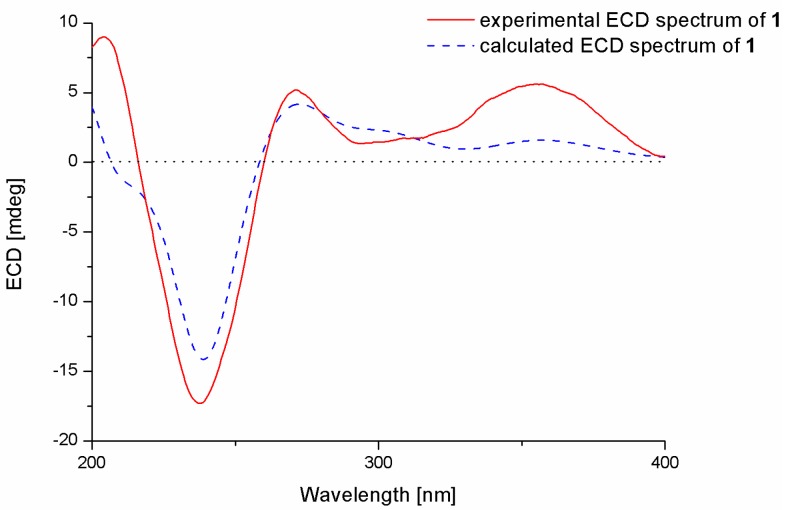
Experimental and calculated ECD spectra of **1**.

To determine the absolute configurations of the stereogenic carbons and of the compound, conformational search, geometry optimization of each possible isomer, and time-dependent density functional (TDDFT)-ECD calculation were performed using Gaussian 09.10. The obtained minimum energy conformers were calculated for their ECD spectra by the TDDFT method at the B3LYP/6-31G(d) level. The experimental ECD spectrum of **1** matched well with that calculated for 3*S*, 4*R*, 7*S*, 9*R*, 13*S*, 16*S*, and 22*R* configurations, which showed positive cotton effects (CEs) at approximately 355 and 272 nm and a negative CE near 237 nm (Figure 4). Thus, the structure of **1** was determined and was named 22-hydroxylshearinine F.

Compound **2** was obtained as a colorless solid. Its molecular formula was determined as C_27_H_31_NO_4_ on the basis of (+)-HRESIMS, indicating 13 degrees of unsaturation. The ^1^H and ^13^C NMR spectra of **2** are very similar to those of paspalinine (Figure 1), an indolediterpenoid isolated from the marine mangrove-derived endophytic fungus *Alternariatenuissima* EN-192 [14]. However, inspection of the NMR data revealed that CH_2_-6 (*δ*_C_ 29.5; *δ*_H_ 2.41/2.64) in paspaline was replaced by an oxymethine group (*δ*_C_ 70.4; *δ*_H_ 4.15) (Table 1). This was further supported by the observed COSY correlation between H-5 and H-6 as well as by the HMBC correlations from H-6 to C-4, C-7, and C-12 (Figure 2).

**Figure 5:**
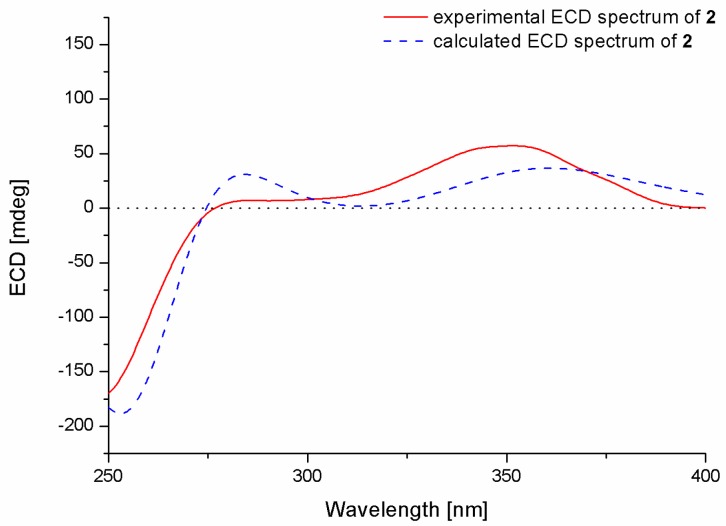
Experimental and calculated ECD spectra of **2**.

The relative configurations of the stereogenic carbons of **2** were determined by NOESY spectrum. The NOESY correlations from H-26 to H-5*β* and H-16 indicated the same orientation of these protons, while the correlations from H-25 to H-5*α* and H-13 and from H-13 to H-28 as well as from H-28 to H-6 and H-9 indicated the opposite orientation of these groups (Figure 3). The absolute configurations of the stereogenic carbons of **2** were also determined by the TDDFT-ECD calculation in Gaussian 09.10. The experimental ECD spectrum of **2** showed excellent agreement with that calculated for 3*S*, 4*S*, 6*R*, 7*R*, 9*R*, 13*R*, and 16*S* configurations (Figure 5), and the structure of **2** was thus established and was named 6-hydroxylpaspalinine.

Compound **3** was obtained as colorless crystals. Its molecular formula was determined as C_30_H_41_NO_2_ on the basis of (+)-HRESIMS data, indicating 11 degrees of unsaturation. Except for the presence of the acetyl group (*δ*_C_ 170.6; *δ*_C_ 21.1/*δ*_H_ 2.05, s), the NMR data of **3** are very similar to those of emindole SB (**7**), an indolediterpenoid isolated from the marine mangrove-derived endophytic fungus *Dichotomomyces* *cejpii* [15], indicating that **3** is an acetylated derivative of **7**. 

**Figure 6:**
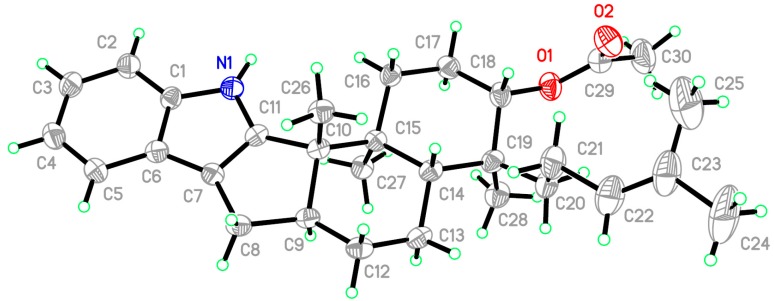
X-ray structure of Compound **3** (note: a different numbering system is used for the structure in the text).

The relative configurations of the stereogenic carbons of **3** were determined by NOESY spectrum. The NOESY correlations from H-21 to H-9 and H-13*α* indicated the same orientation of these protons, while correlations from H-22 to H-12, H-23, and H-31 as well as from H-12 to H-13*β* indicated the other orientation of these protons (Figure 3). X-ray analysis confirmed the structure of **3** and determined the absolute configurations of C-3, 4, 7, 8, and 9 as 3*S*, 4*S*, 7*S*, 8*S*, and 9*S*. The ortep view of **3** is shown in Figure 6. Compound **3** was named 7-*O*-acetylemindole SB.

In addition to the isolation of Compounds **1**–**3**, eight other congeners—shearinine F (**4**) [13], paspalitrem C (**5**) [16], paspalitrem A (**6**) [16], emindole SB (**7**) [15], paspaline (**8**) [17], 3-deoxo-4b-deoxypaxilline (**9**) [18], PC-M6 (**10**) [19], and 10,23-dihydro-24,25-dehydroaflavinine (**11**) [20]—were also isolated and identified. Their structures were determined by analysis of the spectroscopic data and by comparison with that reported in the literature.

### 2.2. Biological Activities of the Isolated Compounds

The isolated compounds (**1**–**11**) were examined for antimicrobial activity against several human-, aqua-, and plant-pathogenic microbes. Compounds **5**, **7**, and **11** showed activity against the aquatic pathogen *Pseudomonas aeruginosa*, with MIC values of 8.0, 1.0, and 0.5 μg/mL, respectively, which are comparable to that of the positive control, chloromycetin (MIC = 0.5 μg/mL). Moreover, Compounds **5**, **7**, **8**, and **11** exhibited activity against the human pathogen *Escherichia coli* with MIC values of 16.0, 4.0, 0.5, and 2.0 μg/mL, respectively, while the positive control chloromycetin has an MIC value of 1.0 μg/mL. Compounds **2**, **5**, **7**, **9**, and **11** exhibited activity against the aquatic pathogen *Vibrio parahaemolyticus* with MIC values of 64.0, 8.0, 2.0, 16.0, and 0.5 μg/mL, respectively, while the positive control chloromycetin has an MIC value of 0.5 μg/mL. Compounds **5**, **7**, and **11** exhibited activity against *V. alginolyticus* with MIC values of 4.0, 1.0, and 0.5 μg/mL, respectively, while the positive control chloromycetin has an MIC value of 0.5 μg/mL.

These data indicated that Compound **5**, with a prenyl group at C-20, has stronger antimicrobial activity than does Compound **6**, where the prenyl substituent is at C-21, while the results from Compounds **8**, **9**, and **10** showed that the double bond at C-11 and the hydroxyl substitute at C-10 significantly influenced the activity. Moreover, acetylation of OH at C-7 decreased the antibacterial activity (**3** vs. **7**).

## 3. Experimental Section

### 3.1. General

Melting points were determined with an SGW X-4 micro-melting-point apparatus. Optical rotations were measured on an Optical Activity AA-55 polarimeter (Optical Activity Ltd., Cambridgeshire, UK). UV spectra were measured on a Lengguang Gold S54 spectrophotometer (Shanghai Lengguang Technology Co. Ltd., Shanghai, China). ECD spectra were acquired on a JASCO J-715 spectropolarimeter (JASCO, Tokyo, Japan). The ^1^H, ^13^C, and 2D NMR spectra were acquired using Bruker Avance 500 spectrometers (Bruker Biospin Group, Karlsruhe, Germany). Mass spectra were determined on a VG Autospec 3000 (VG Instruments, London, UK) or an API QSTAR Pulsar 1 mass spectrometer (Applied Biosystems, Foster, Waltham, MA, USA). Analytical HPLC and semi-preparative HPLC were performed using a Dionex HPLC system equipped with a P680 pump, an ASI-100 automated sample injector, and a UVD340U multiple wavelength detector controlled by Chromeleon software (version 6.80) (Dionex, Sunnyvale, CA, USA). Column chromatography (CC) was performed with silica gel (200–300 mesh, Qingdao Haiyang Chemical Factory, Qingdao, China), Lobar LiChroprep RP-18 (40–60 μm, Merck, Darmstadt, Germany), and Sephadex LH-20 (18–110 μm, Merck).

### 3.2. Fungal Material

The fungus *Penicillium* sp. AS-79 was isolated from the fresh tissue of the sea anemone *Haliplanella luciae*, which was collected from the Qingdao coastline in December 2014. The fungus was identified by sequence analysis of the ITS [21] and beta-tubulin [22] region of its rDNA as described previously. The resulting sequence data obtained from the fungal strain have been deposited in GenBank (with accession No. KY322516). A BLAST search result indicated that the ITS sequence was most similar (99%) to the sequence of *Penicillium* *janthinellum* (compared to KM268705.1), but the beta-tubulin sequence was most similar (95%) to *P. vasconiae* (compared to KT887805.1). Therefore, it is hard to identify the strain to a species level. The strain is preserved at the key Laboratory of Experimental Marine Biology, Institute of Oceanology, Chinese Academy of Sciences.

### 3.3. Fermentation

The fermentation was carried out statically on a rice solid medium (each flask contained 70 g of rice (Cofco, Beijing, China), 0.1 g of corn flour (Macklin, Shanghai, China), 0.3 g of peptone (Shuangxuan, Beijing, China), 0.1 g of sodium glutamate (Lianhua, Henan, China), and 100 mL of naturally sourced and filtered seawater, which was obtained from the Huiquan Gulf of the Yellow Sea near the campus of the authors’ institution, pH 6.5–7.0) in 1 L Erlenmeyer flasks for 30 days at room temperature.

### 3.4. Extraction and Isolation

The whole fermented broth (66 flasks) was extracted exhaustively with EtOAc, yielding 72.5 g of crude extract, which was subjected to silica gel vacuum liquid chromatography (VLC) eluting with mixed solvents of increasing polarity (petroleum ether (PE) to MeOH) to yield nine fractions (Fraction 1 to Fraction 9). Fraction 3 (5.1 g) was further purified by reverse-phase column chromatography (CC) over Lobar LiChroprep RP-18 with a MeOH–H_2_O gradient (from 20:80 to 100:0) to afford two subfractions (Fraction 3.1 and Fraction 3.2). Fraction 3.2 was further purified by prep. TLC (plate: 20 × 20 cm, developing solvents: PE/acetone, 10:1), then by CC on Sephadex LH-20 (MeOH), and finally by prep. TLC (plate: 20 × 20 cm, developing solvents: CH_2_Cl_2_/MeOH, 40:1) to obtain Compounds **3** (71.8 mg) and **11** (12.4 mg). Fraction 4 (2.0 g) was further purified by CC on silica gel (eluted with CH_2_Cl_2_-MeOH, 200:1 to 60:1), then on Sephadex LH-20 (MeOH) and finally on silica gel (eluted with CH_2_Cl_2_) to obtain Compound **6** (11.8 mg). Fraction 5 (5.9 g) was further purified by CC over Lobar LiChroprep RP-18 with a MeOH–H_2_O gradient (from 20:80 to 100:0) to afford eight subfractions (Fraction 5.1–Fraction 5.8). Fraction 5.4 and Fraction 5.5 was further purified by CC on silica gel (eluted with CH_2_Cl_2_-MeOH, 100:1 to 60:1). Then Fraction 5.4 was purified by prep. TLC (plate: 20 × 20 cm, developing solvents: CH_2_Cl_2_/MeOH, 40:1) to obtain Compound **1** (12.3 mg). Fraction 5.5 (3.5 g) was further purified by CC on Sephadex LH-20 (MeOH) to obtain Compound **2** (5.2 mg). Fraction 5.7 (48.9 mg) was further purified by CC on silica gel (eluted with CH_2_Cl_2_-MeOH, 200:1 to 100:1) to obtain Compounds **4** (5.8 mg) and **8** (8.9 mg). Fraction 5.8 (139 mg) was further purified by CC on silica gel (eluted with CH_2_Cl_2_-MeOH, 200:1 to 100:1) and by prep. TLC (plate: 20 × 20 cm, developing solvents: CH_2_Cl_2_/MeOH, 80:1) to obtain Compounds **5** (10.0 mg) and **7** (9.7 mg). Fraction 6 (5.8 g) was further purified by CC over Lobar LiChroprep RP-18 with a MeOH–H_2_O gradient (from 20:80 to 100:0) to afford five subfractions (Fraction 6.1–Fraction 6.5). Fraction 6.4 (100.0 mg) was further purified by CC on silica gel (eluted with PE-EtOAc, 10:1 to 8:1) to obtain Compound **9** (4.9 mg). Fraction 6.5 was further purified by prep. HPLC (MeOH–H_2_O, 85:15) to obtain Compound **10** (11.2 mg, *t*_R_ 13.0 min).

22-Hydroxylshearinine F (**1**): colorless solid; ${\left[\alpha \right]}_{D}^{20}$: +50 (*c* 0.1, MeOH); UV (MeOH) λ_max_ (log ε) 215 (4.39), 251 (4.29), 317 (4.02) nm; ECD λ_max_ (Δε) 237.5 (−12.18), 272 (+3.54) nm, ^1^H and ^13^C NMR data, see Table 1; ESIMS *m/z* 600 [M + H]^+^; HRESIMS *m/z* 600.3330 [M + H]^+^ (calcd. for C_37_H_46_NO_6_, 600.3320).

6-Hydroxylpaspalinine (**2**): yellowish solid; ${\left[\alpha \right]}_{D}^{20}$: +30.8 (*c* 0.13, MeOH); UV (MeOH) λ_max_ (log ε) 221 (3.51), 232 (3.49) nm; ECD λ_max_ (Δε) 250 (−9.79), 354.5 (+30.62) nm;^1^H and ^13^C NMR data, see Table 1; ESIMS *m/z* 434 [M + H]^+^; HRESIMS *m/z* 434.2332 [M + H]^+^ (calcd. for C_27_H_32_NO_4_, 434.2326).

7-*O*-Acetylemindole SB (**3**): colorless crystals; m.p. 192–194 °C; ${\left[\alpha \right]}_{D}^{20}$: +45.5 (*c* 0.11, MeOH); UV (MeOH) λ_max_ (log ε) 229 (3.80), 281 (3.17) nm; ECD λ_max_ (Δε) 250 (+6.50), 295 (−2.79) nm; ^1^H and ^13^C NMR data, see Table 1; ESIMS *m/z* 448 [M + H]^+^; HRESIMS *m/z* 448.3207 [M + H]^+^ (calcd. for C_30_H_42_NO_2_, 448.3210).

### 3.5. X-ray Crystallographic Analysis of Compound ***3***

All crystallographic data were collected on an Agilent Xcalibur Eos Gemini CCD plate diffractometer (Agilent Technologies, Santa Clara, CA, USA), using graphite monochromatized Cu K*α* radiation (λ = 1.54178 Å) for **3** [23]. The data were corrected for absorption by using the program SADABS [24]. The structures were solved by direct methods with the SHELXTL software package (Sheldrick G.M., University of Göttingen, Germany) [25]. All non-hydrogen atoms were refined anisotropically. The H atoms were located by geometrical calculations, and their positions and thermal parameters were fixed during the structure refinement. The structure was refined by full-matrix least-squares techniques [26].

*Crystal data for compound* **3**: C_30_H_41_NO_2_, F.W. = 447.64, orthorhombic space group, P2(1)2(1)2(1), unit cell dimensions *a* = 6.8164(4) Å, *b* = 7.6058(6) Å, *c* = 50.959(3) Å, *V* = 2641.9(3) Å^3^, α = β = γ = 90°, *Z* = 4, *d*_calcd_ = 1.125 mg/m^3^, crystal dimensions 0.40 × 0.33 × 0.14 mm, *μ* = 0.531 mm^−^^1^, *F*(000) = 976. The 4587 measurements yielded 306 independent reflections after equivalent data were averaged, and Lorentz and polarization corrections were applied. The final refinement gave *R*_1_ = 0.0641 and w*R*_2_ = 0.1236 [*I* > 2σ(*I*)]. The final refinement Flack parameter is 0.0(5).

### 3.6. Antimicrobial Assay

Antimicrobial assay against human- and aqua-pathogenic microbes *Edwardsiella tarda*, *Escherichia* *coli*, *Micrococcus luteus*, *Pseudomonas aeruginosa*, *Vibrio alginolyticus*, *V. harveyi*, *V. parahaemolyticus*, and plant pathogenic fungi *Alternaria alternata*, *A.* *brassicae*, *Colletotrichum gloeosprioides*, *Fusarium graminearum*, *F. oxysporum*, *Gaeumannomyces* *graminis*, *Phytophthora nicotiana*, *Physalospora piricola*, and *Valsa mali* was carried out using the well diffusion method [27]. Chloromycetin was used as a positive control for the bacteria, while amphotericin B was used as a positive control for the fungi.

## 4. Conclusions

Three new indolediterpenoid derivatives (**1**–**3**), along with eight known analogs (**4**–**11**), were isolated from the sea anemone-derived fungus *Penicillium* sp. AS-79. The structures and relative configurations were determined via the interpretation of NMR data, and the absolute configurations of all compounds were determined via ECD comparison, while the structure of Compound **3** was confirmed by single-crystal X-ray diffraction analysis. Several compounds exhibited activity against some of the tested microbial strains.

## Figures and Tables

**Table 1:** ^1^H (125 MHz) and ^13^C NMR (500 MHz) data of Compounds **1**–**3** (*δ* in ppm, *J* in Hz).

No.	1 *^a^*		2 *^a^*		3 *^b^*	
	^1^H	^13^C	^1^H	^13^C	^1^H	^13^C
1	7.75, s		7.73, s		9.79, s	
2		151.8, C		149.9, C		151.8, C
3		51.8, C		51.2, C		53.9, C
4		40.1, C		38.1, C		38.6, C
5	*α* 2.79, m	27.2, CH_2_	*α* 2.59, d (14.8)	37.5, CH_2_	*α* 1.80, m	24.7, CH_2_
	*β* 1.72, m		*β* 2.17, d (5.2)		*β* 1.73, m	
6	*α* 2.82, m	28.4, CH_2_	4.15, d (5.0)	70.4, CH	α 1.89, m	23.3, CH_2_
	*β* 2.05, m				*β* 1.69, m	
7		104.5, C		103.3, C	4.88, dd (10.7, 4.3)	75.9, CH
8						40.0, C
9	4.33, s	88.2, CH	4.37, s	88.4, CH	1.51, br.s	41.0, CH
10		197.2, C		195.2, C	*α* 1.79, m	28.0, CH_2_
					*β*1.72, m	
11	5.86, s	117.8, CH	5.87, s	118.7, CH	*α* 1.80, m	33.1, CH_2_
					*β* 1.68, m	
12		169.8, C		171.3, C	2.80, m	49.8, CH
13		77.8, C	3.68, dt (3.4, 11.7)	39.5, CH	*α* 2.36, dd (13.1, 10.9)	28.0, CH_2_
					*β* 2.68, dd (13.1, 6.4)	
14	*α* 2.05, m	34.5, CH_2_	*α* 1.97, m	24.1, CH_2_		117.9, C
	*β* 1.92, m		*β* 1.48, m			
15	*α* 2.05, m	21.3, CH_2_	*α* 1.85, m	24.0, CH_2_		131.5, C
	*β* 1.72, m		*β* 1.77, m			
16	2.64, m	48.6, CH	2.83, m	48.4, CH	7.36, d (7.4)	118.8, CH
17	*α* 2.79, dd (12.4, 5.6)	27.8, CH_2_	*α* 2.74, dd (13.2, 6.3)	27.8, CH_2_	6.99, dt (7.1, 1.6)	119.7, CH
	*β* 2.05, dd (12.4, 3.1)		*β* 2.41, dd (13.2, 10.6)			
18		117.8, C		118.5. C	6.99, dt (7.1, 1.6)	120.6, CH
19		123.6, C		125.3, C	7.32, d (7.3)	112.6, C
20	7.56, s	114.1, CH	7.46, d (7.9)	119.9, CH	7.32, d (7.3)	141.7, C
21		136.8, C	7.11, t (6.2)	120.8, CH	1.10, s	15.0, CH_3_
22	5.19, s	76.3, CH	7.11, t (6.2)	121.5, CH	1.18, s	19.4, CH_3_
23		144.8, C	7.34, d (8.2)	111.7, CH	0.95, s	18.1, CH_3_
24		73.8, C		140.3, C	*α* 1.52, m	29.9, CH_2_
					*β* 1.23, m	
25			1.16, s	15.5, CH_3_	*α* 2.08, m	22.1, CH_2_
					*β* 1.80, m	
2*6*		71.3, C	1.18, s	21.6, CH_3_	5.11, t (7.1)	125.5, CH
27	*α* 2.45, d (18.0)	34.1, CH_2_		79.8, C		126.1, C
	*β* 2.43, d (18.0)					
28		138.4, C	1.44, s	28.7, CH_3_	1.63, s	25.9, CH_3_
29		133.9, C	1.24, s	23.2, CH_3_	1.69, s	17.6, CH_3_
30	7.02, s	102.1, CH				170.6, C
31		140.0, C			2.05, s	21.1, CH_3_
32	1.37, s	16.3, CH_3_				
33	1.25, s	23.8, CH_3_				
34		78.9, C				
35	1.20, s	23.2, CH_3_				
36	1.46, s	29.0, CH_3_				
37	1.55, s	30.4, CH_3_				
38	1.50, s	30.0, CH_3_				
39	1.29, s	29.7, CH_3_				
40	1.37, s	29.5, CH_3_				

*^a^* Measured in CDCl_3_. *^b^* Measured in acetone-*d*_6_.

## Supplementary Materials

The following are available online at www.mdpi.com/1660-3397/15/5/137/s1. 1D and 2D NMR spectra of Compounds **1**–**3** as well as crystal packing of Compound **3**.
