# Supplementary material for: Three New Indole Diterpenoids from the Sea-Anemone-Derived Fungus Penicillium sp. AS-79
Source: Mar Drugs. 2017 May 12;15(5):137. doi: 10.3390/md15050137 (PMC5450543; doi:10.3390/md15050137)

Supplementary Material

Three New Indole Diterpenoids from the Sea-Anemone-Derived Fungus *Penicillium* sp. AS-79

Xue-Yi Hu [1,2], Ling-Hong Meng [1], Xin Li [1], Sui-Qun Yang [1,2], Xiao-Ming Li [1] and Bin-Gui Wang [1,*]

[1] Laboratory of Marine Biology and Biotechnology, Qingdao National Laboratory for Marine Science and Technology, Key Laboratory of Experimental Marine Biology, Institute of Oceanology, Chinese Academy of Sciences, Nanhai Road 7, Qingdao 266071, China; E-Mails: huxueyi14@mails.ucas.ac.cn (X.-Y.H.); m8545303@163.com (L.-H.M.); lixin871014@163.com (X.L.); suiqunyang@163.com (S.-Q.Y.); lixmqdio@126.com (X.-M.L.)

[2] College of Earth Science, University of Chinese Academy of Sciences, Yuquan Road 19A, Beijing 100049, China

***** Correspondence: wangbg@ms.qdio.ac.cn;
Tel./Fax: +86-532-8289-8553.

Contents

S1. ^{1}H NMR (500 MHz, $CDCl_3$) spectrum of compound **1**;

S2. ^{13}C NMR (125 MHz, $CDCl_3$) and DEPT spectra of compound **1**;

S3. ^{1}H-^{1}H COSY spectrum of compound **1**;

S4. HSQC (600 MHz, $CDCl_3$) spectrum of compound **1;**

S5. HMBC spectrum of compound **1**;

S6. NOESY spectrum of compound **1**;

S7. ^{1}H NMR (500 MHz, $CDCl_3$) spectrum of compound **2**;

S8. ^{13}C NMR (125 MHz, $CDCl_3$) and DEPT spectra of compound **2**;

S9. ^{1}H-^{1}H COSY spectrum of compound **2**;

S10. HMBC spectrum of compound **2**;

S11. NOESY spectrum of compound **2**;

S12. ^{1}H NMR (500 MHz, acetone-d_6) spectrum of compound **3**;

S13. ^{13}C NMR (125 MHz, acetone-d_6) and DEPT spectra of compound **3**;

S14. ^{1}H-^{1}H COSY spectrum of compound **3**;

S15. HSQC spectrum of compound **3**;

S16. HMBC spectrum of compound **3**;

S17. NOESY spectrum of compound **3**;

S18. Crystal packing of compound **3** at 293(2) K.

S1. ^{1}H NMR (500 MHz, $CDCl_3$) spectrum of compound **1**.

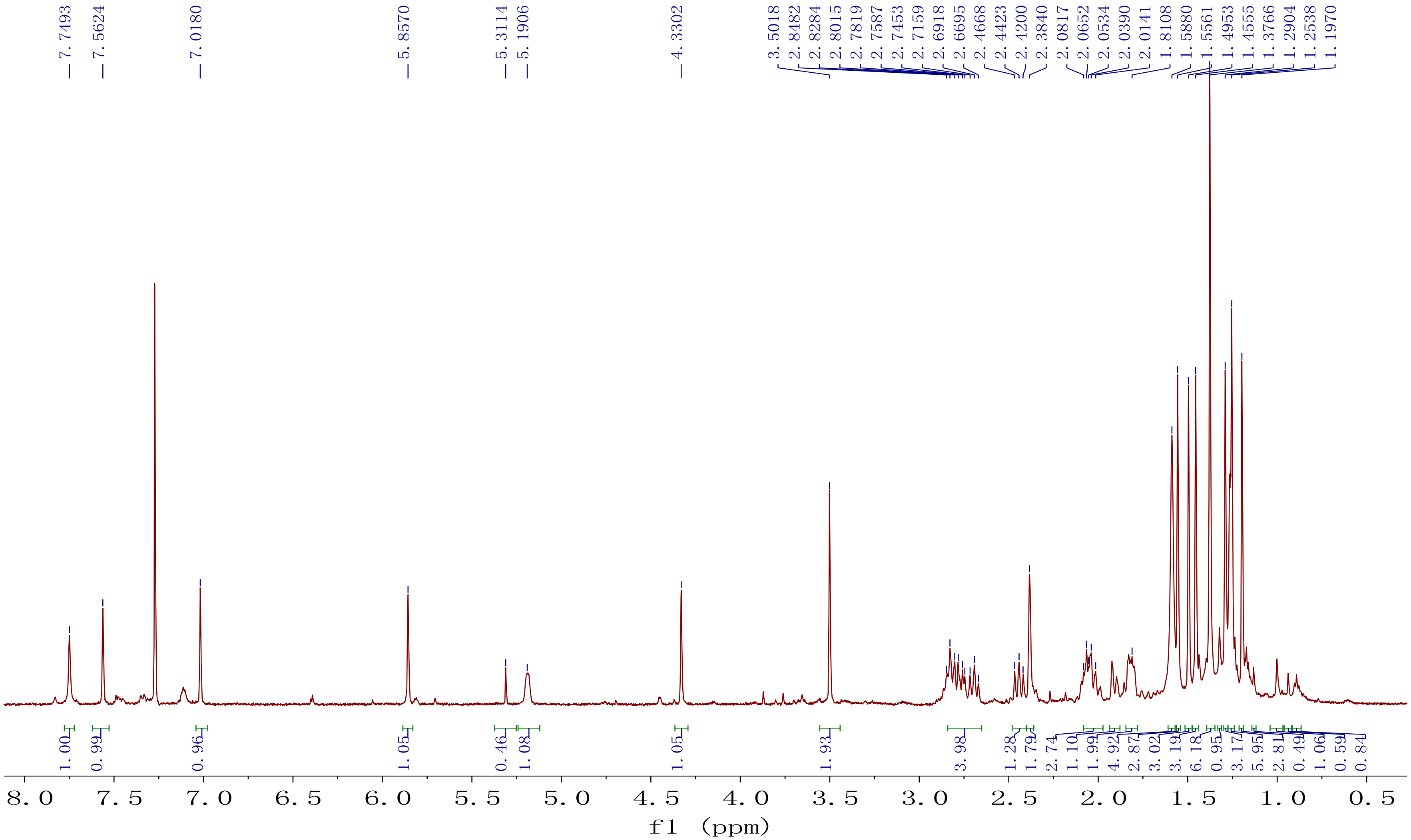

S2. ^{13}C NMR (125 MHz, $CDCl_3$) and DEPT spectra of compound **1**.

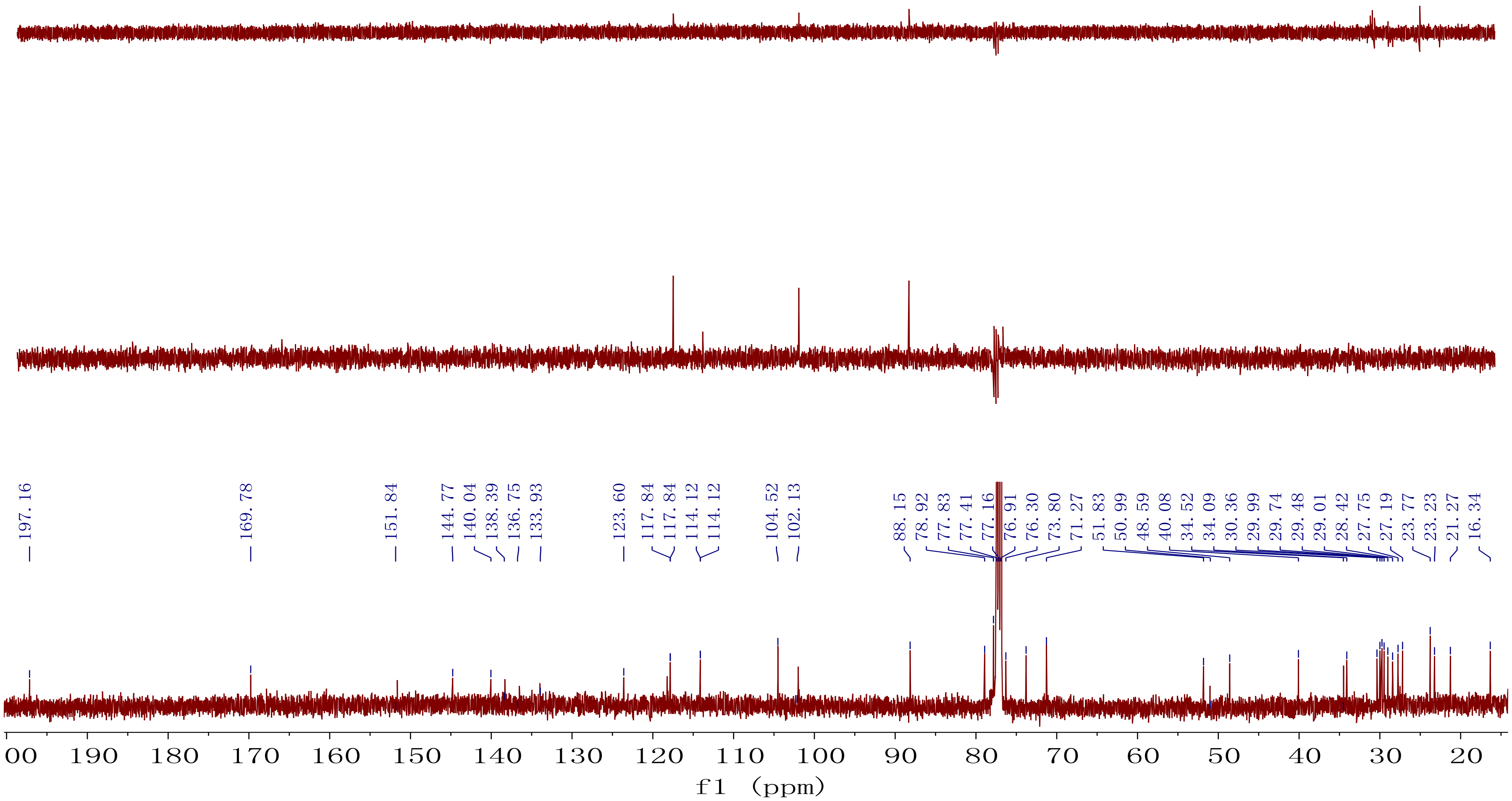

S3. ^{1}H-^{1}H COSY spectrum of compound **1**.

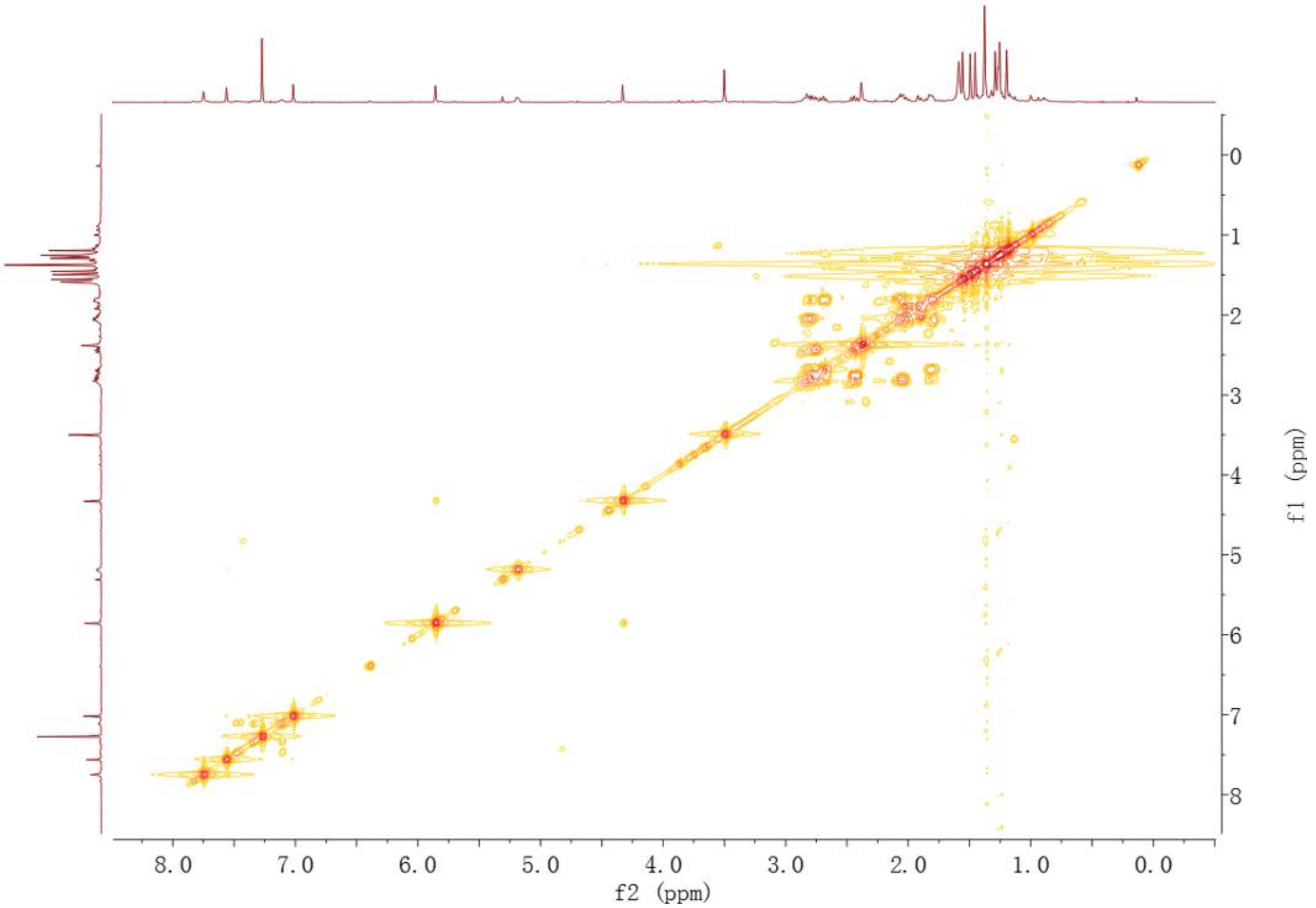

S4. HSQC (600 MHz, $CDCl_3$) spectrum of compound **1**

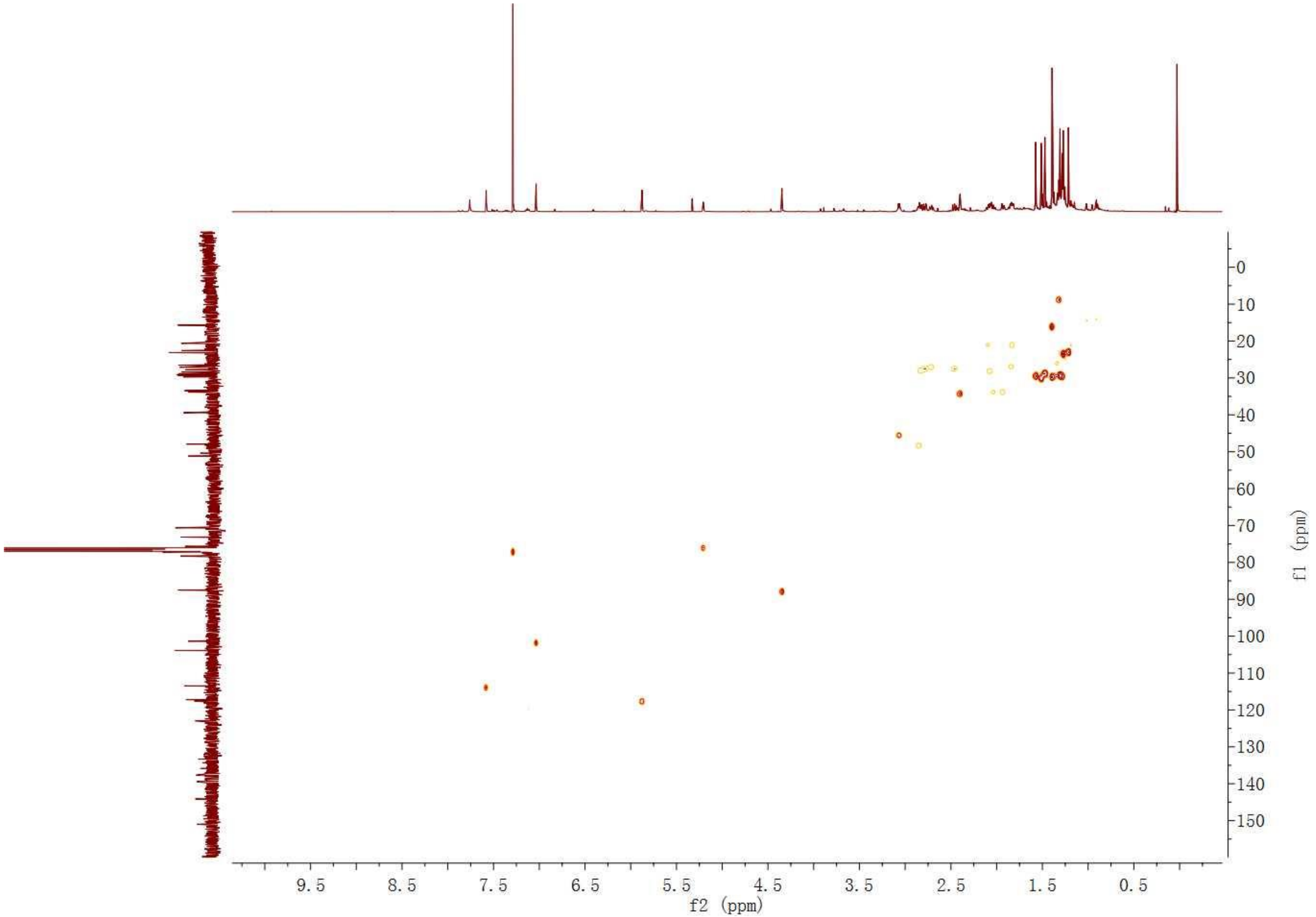

S5. HMBC spectrum of compound **1**.

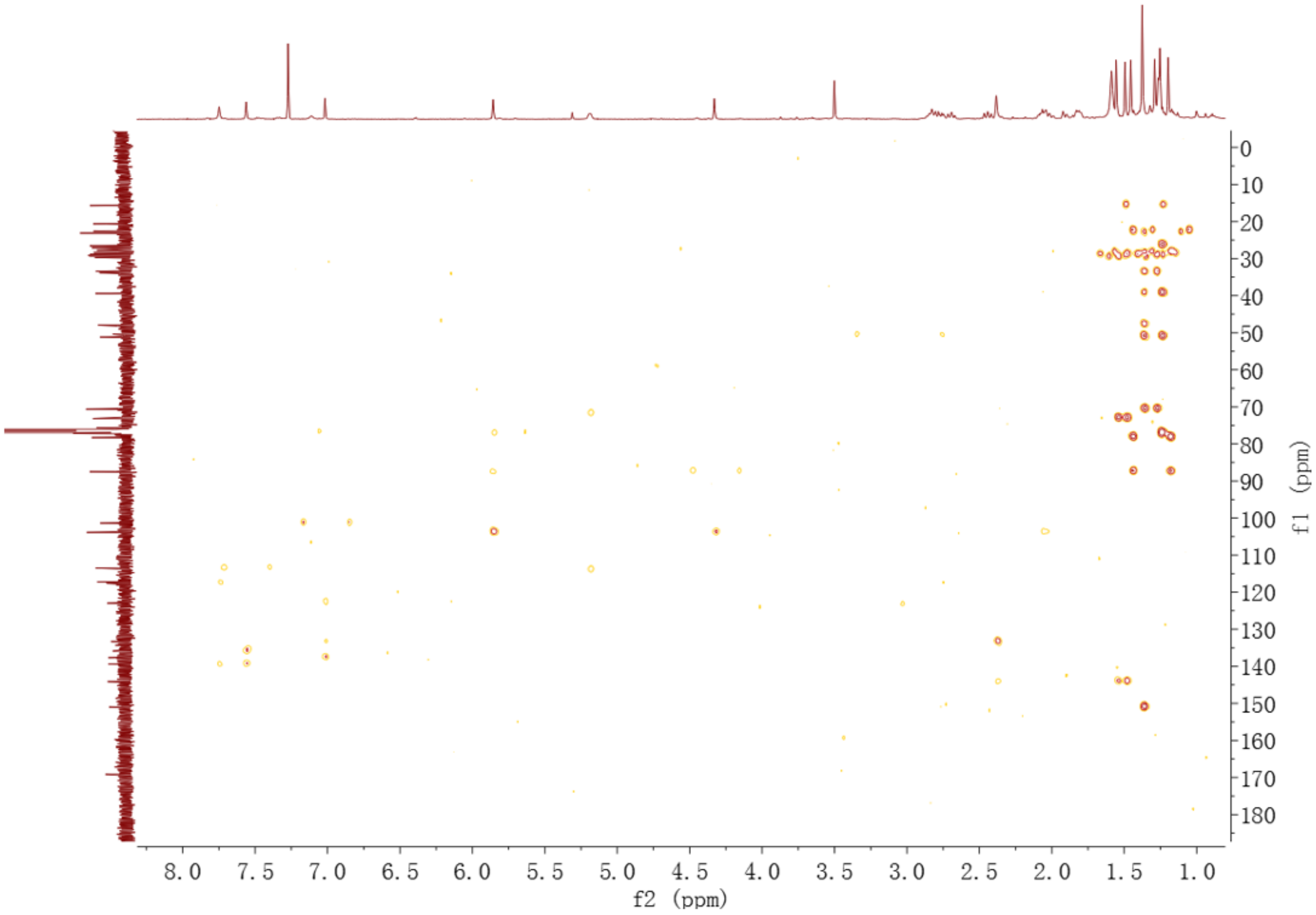

S6. NOESY spectrum of compound **1**.

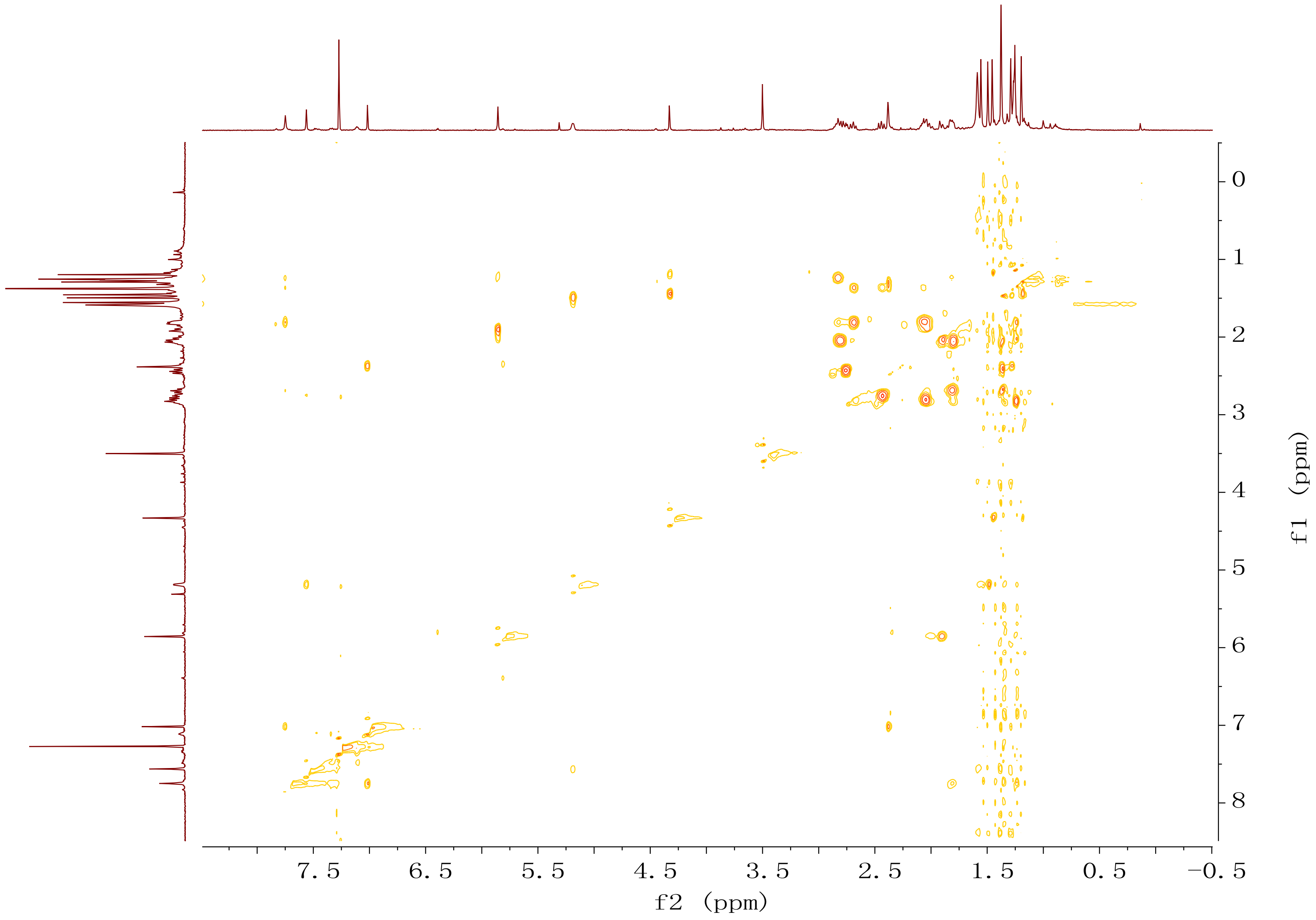

S7. ^{1}H NMR (500 MHz, $CDCl_3$) spectrum of compound **2**.

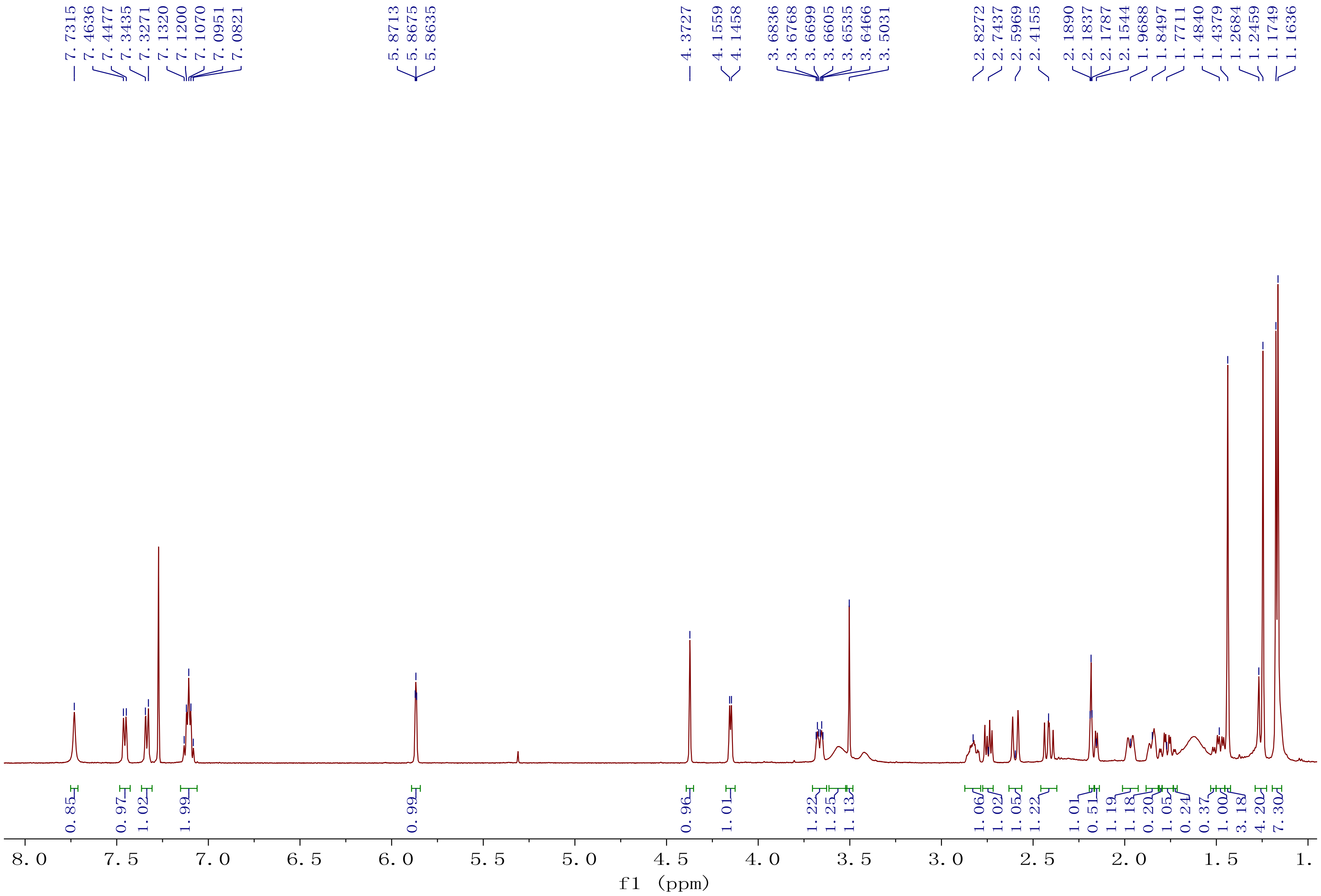

S8. ^{13}C NMR (125 MHz, $CDCl_3$) and DEPT spectra of compound **2**.

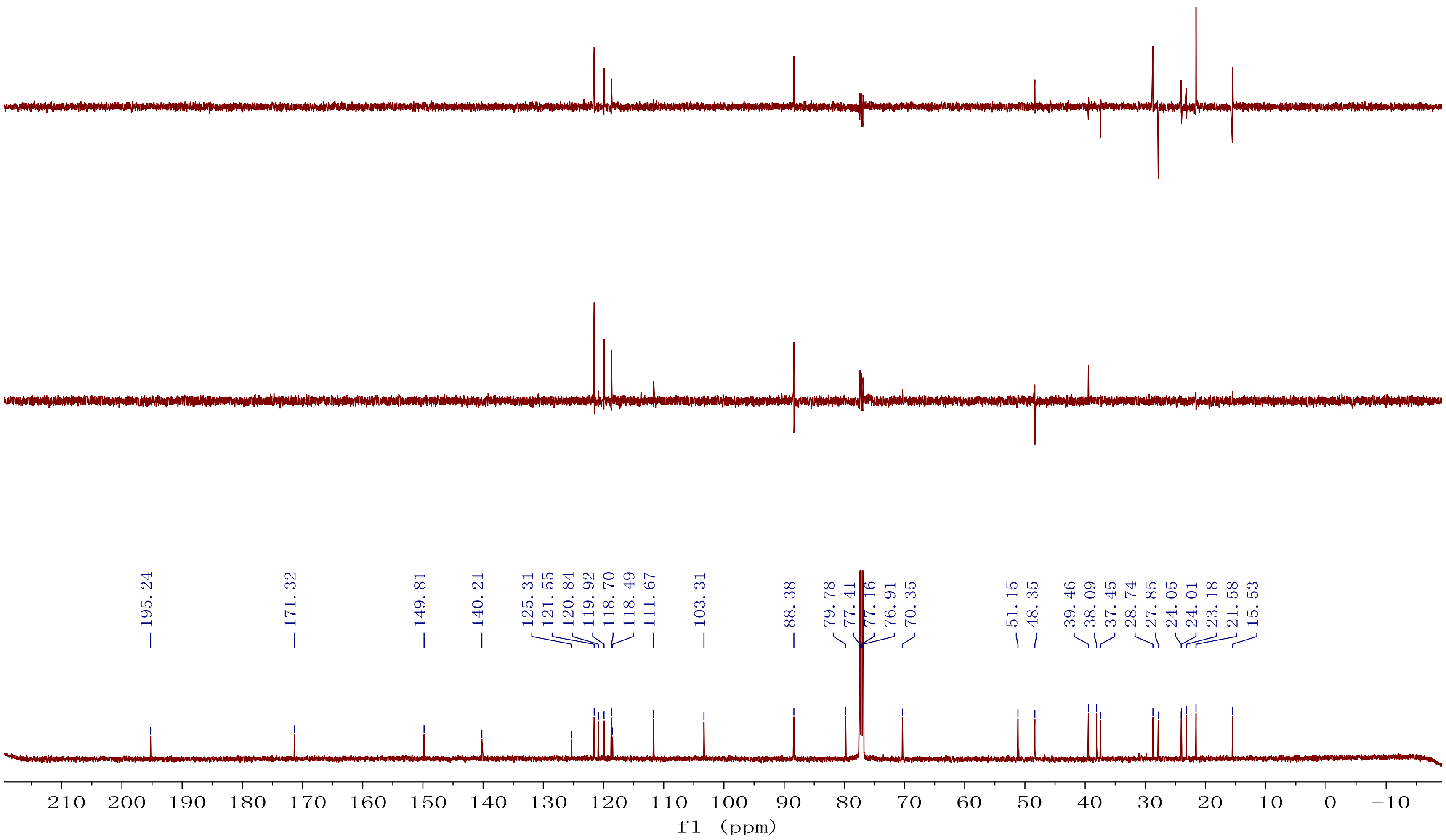

S9. ^{1}H-^{1}H COSY spectrum of compound **2**.

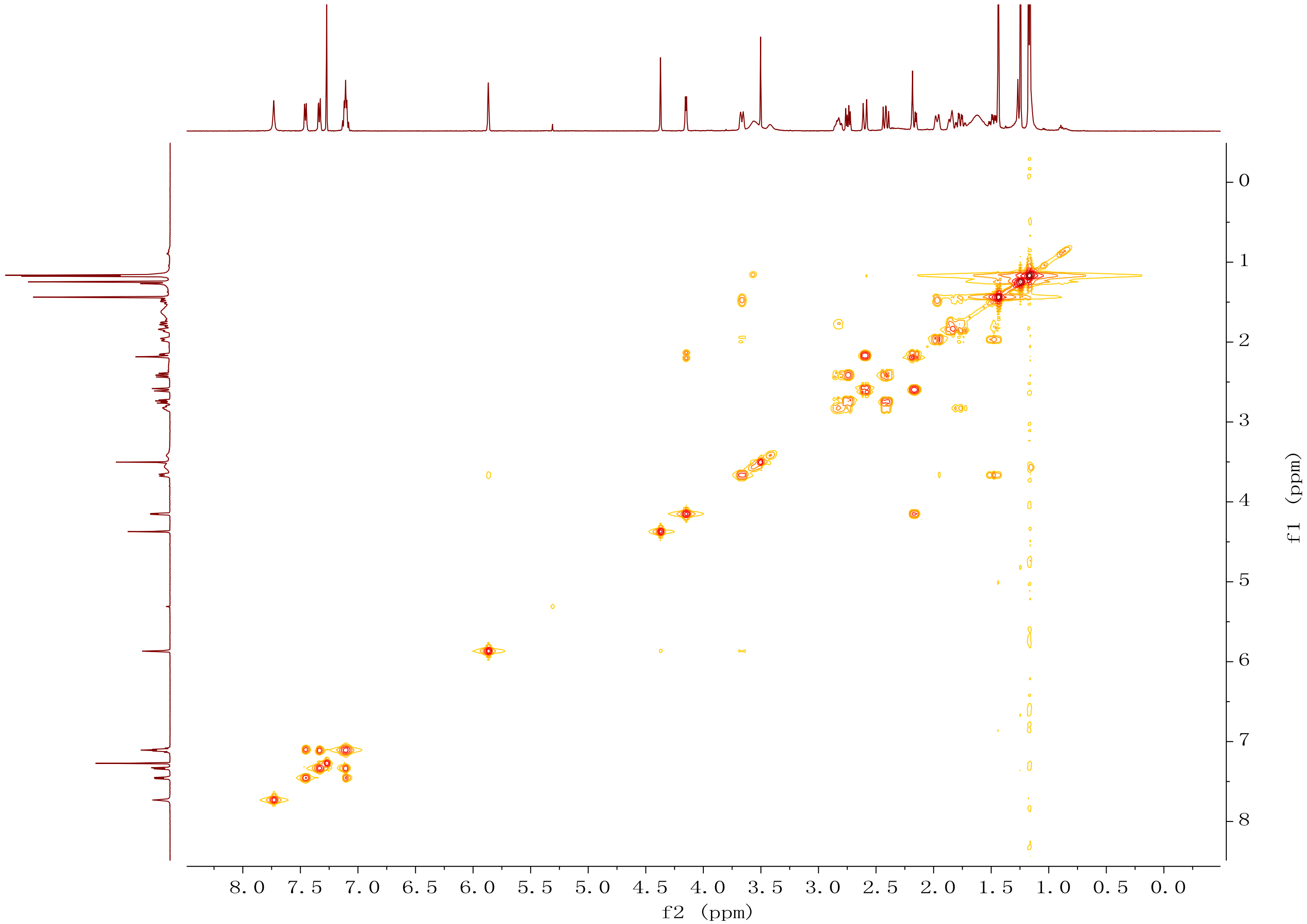

.

S10. HMBC spectrum of compound **2**.

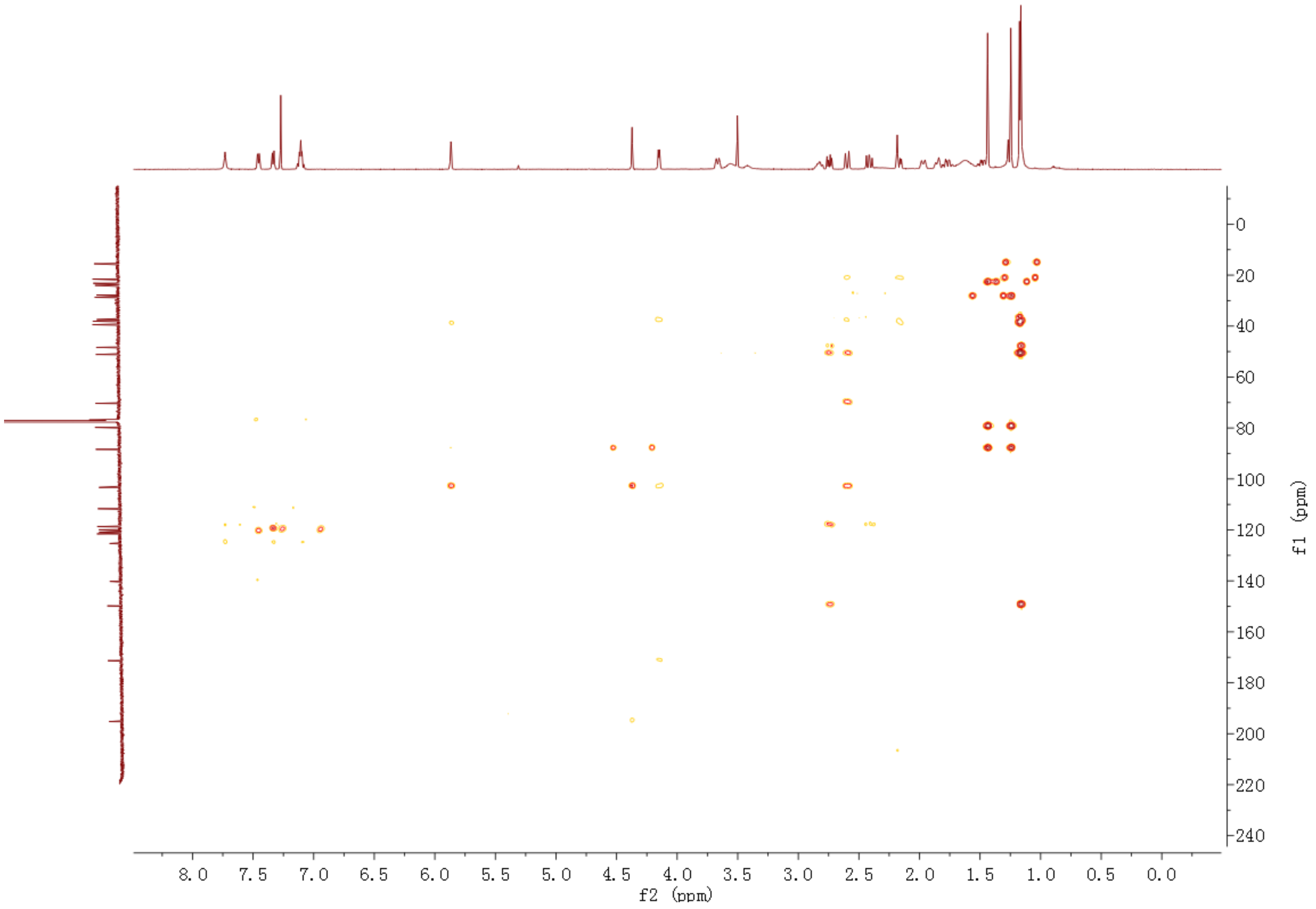

S11. NOESY spectrum of compound **2**.

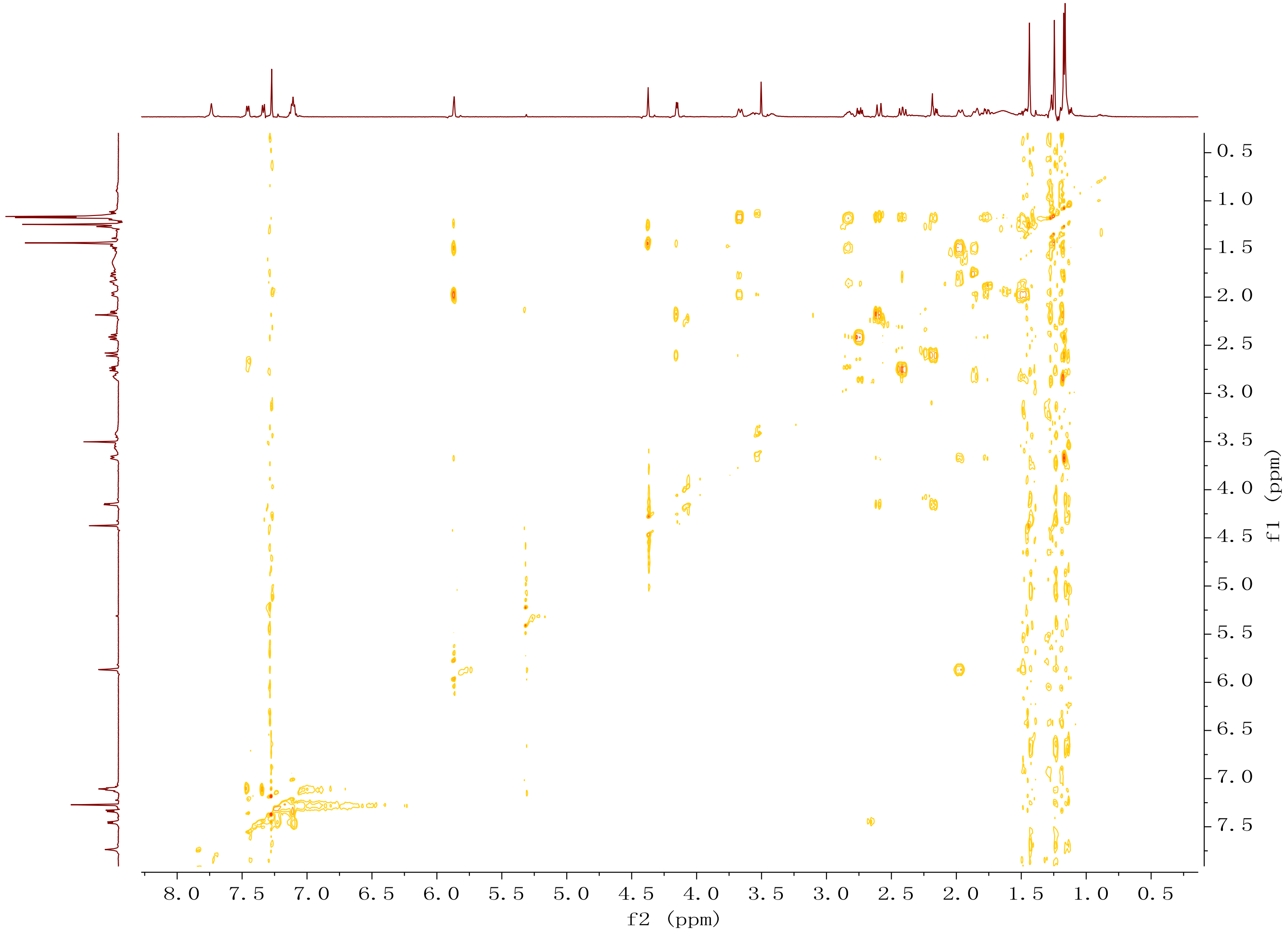

S12. ^{1}H NMR (500 MHz, acetone-d_6) spectrum of compound **3**.

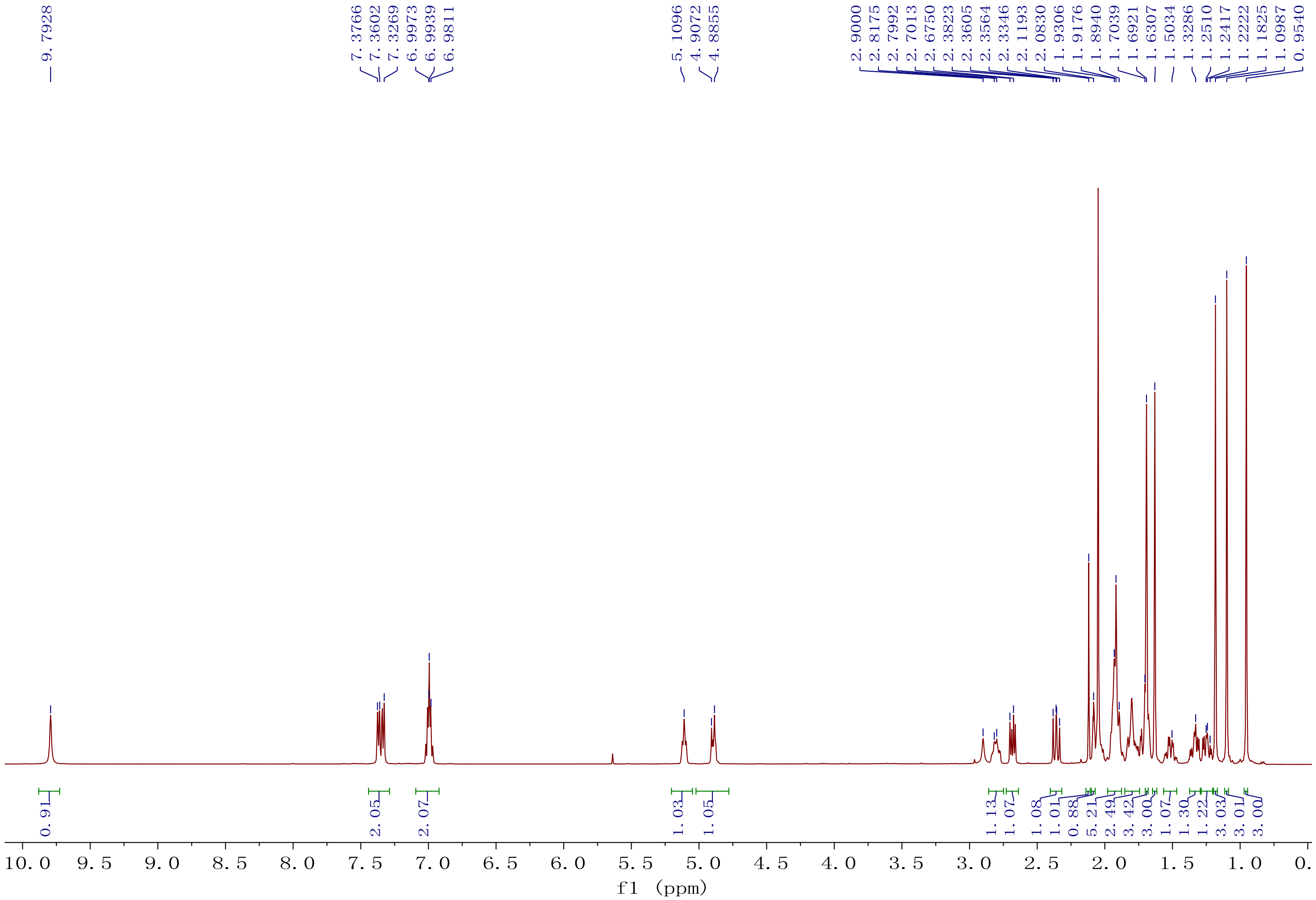

S13. ^{13}C NMR (125 MHz, acetone-d6) and DEPT spectra of compound **3.**

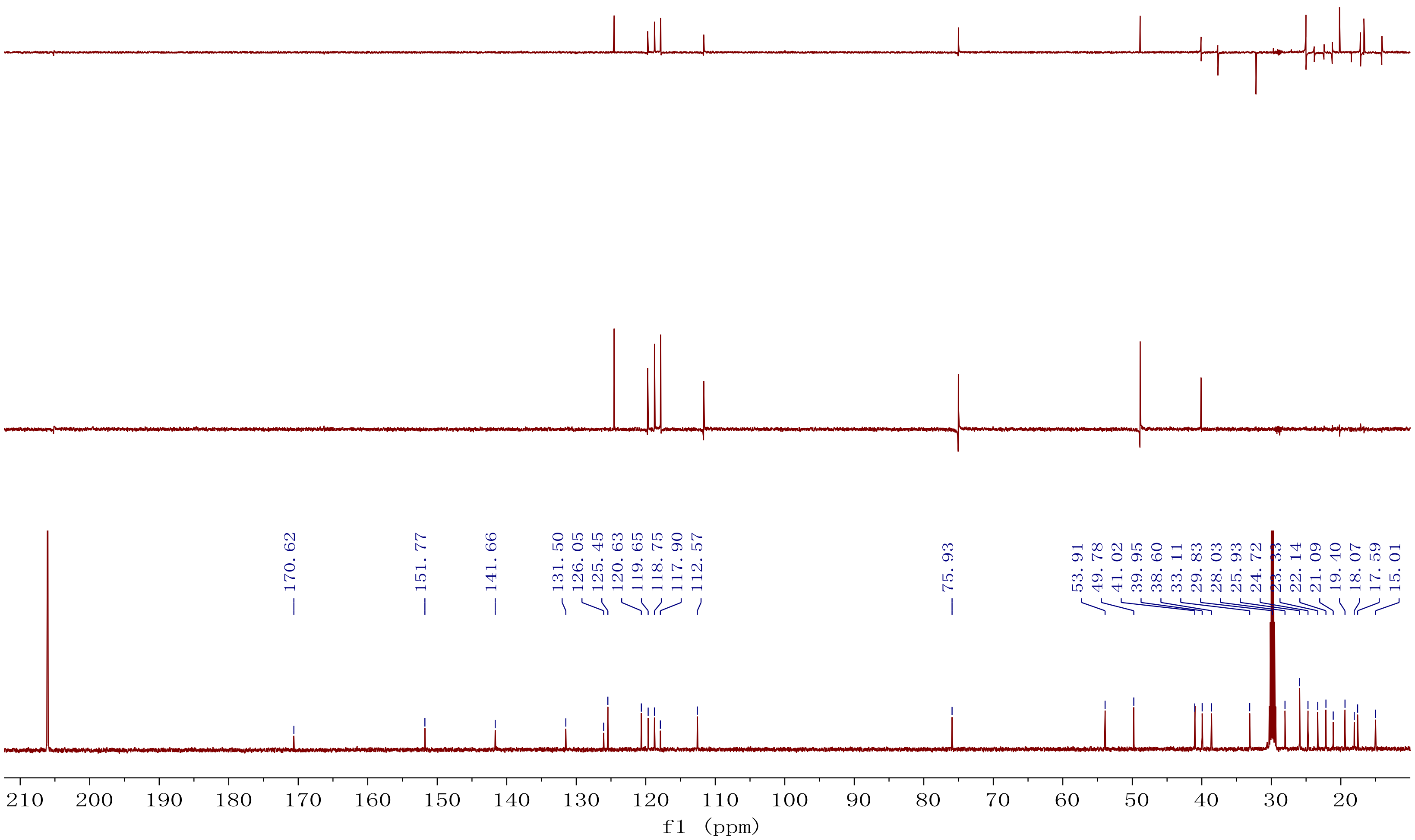

S14. ^{1}H-^{1}H COSY spectrum of compound **3**.

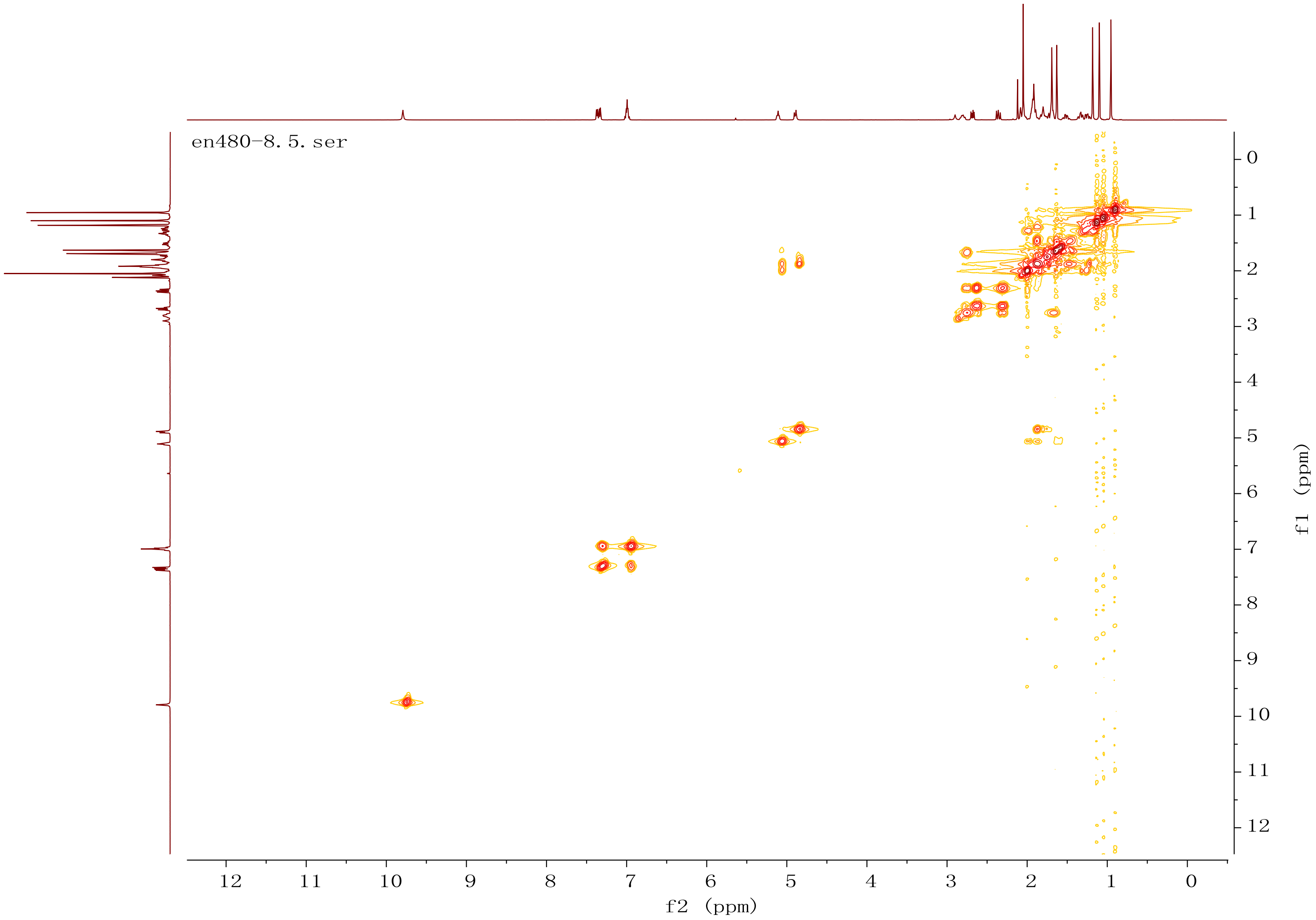

S15. HSQC spectrum of compound **3**.

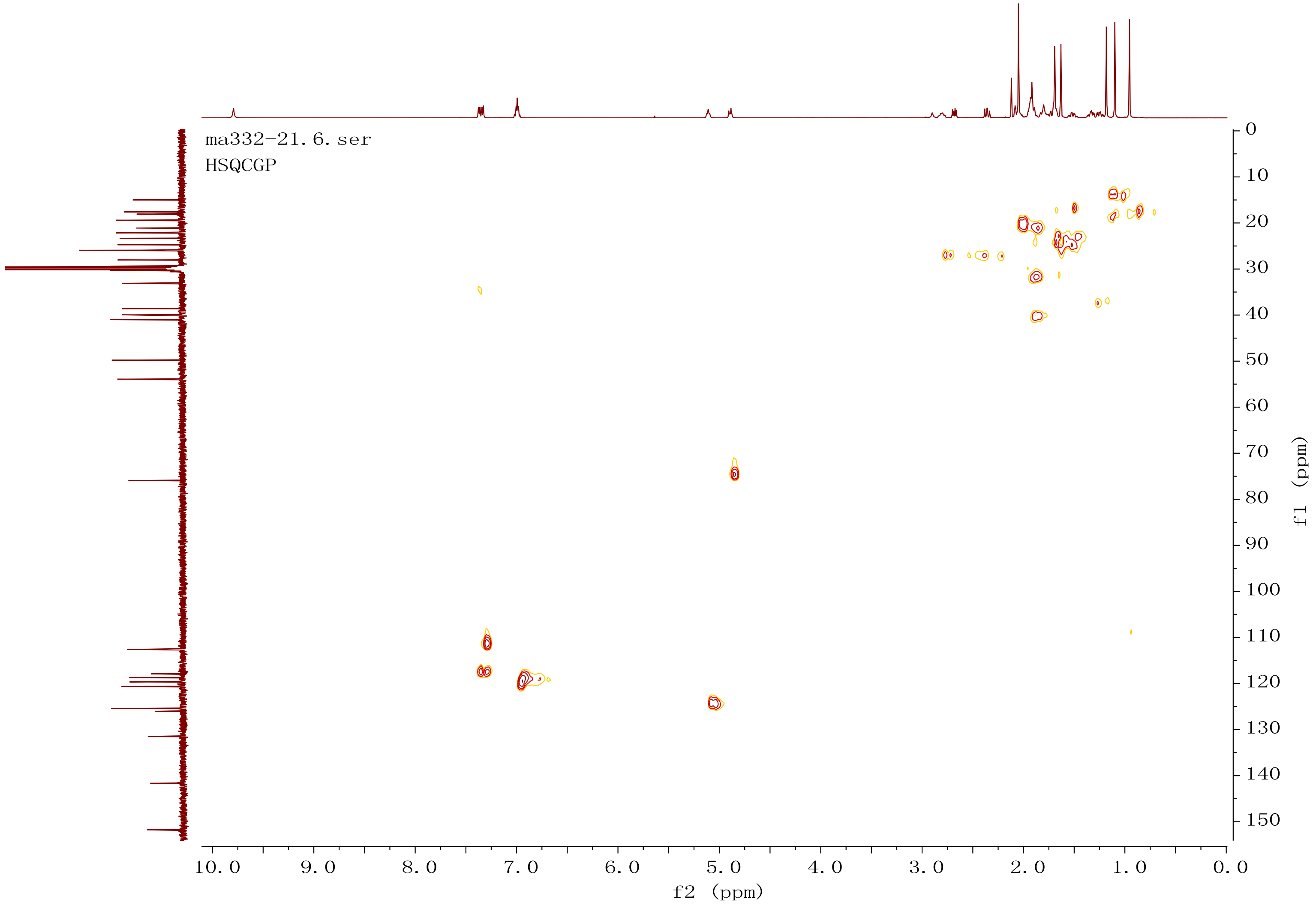

S16. HMBC spectrum of compound **3**.

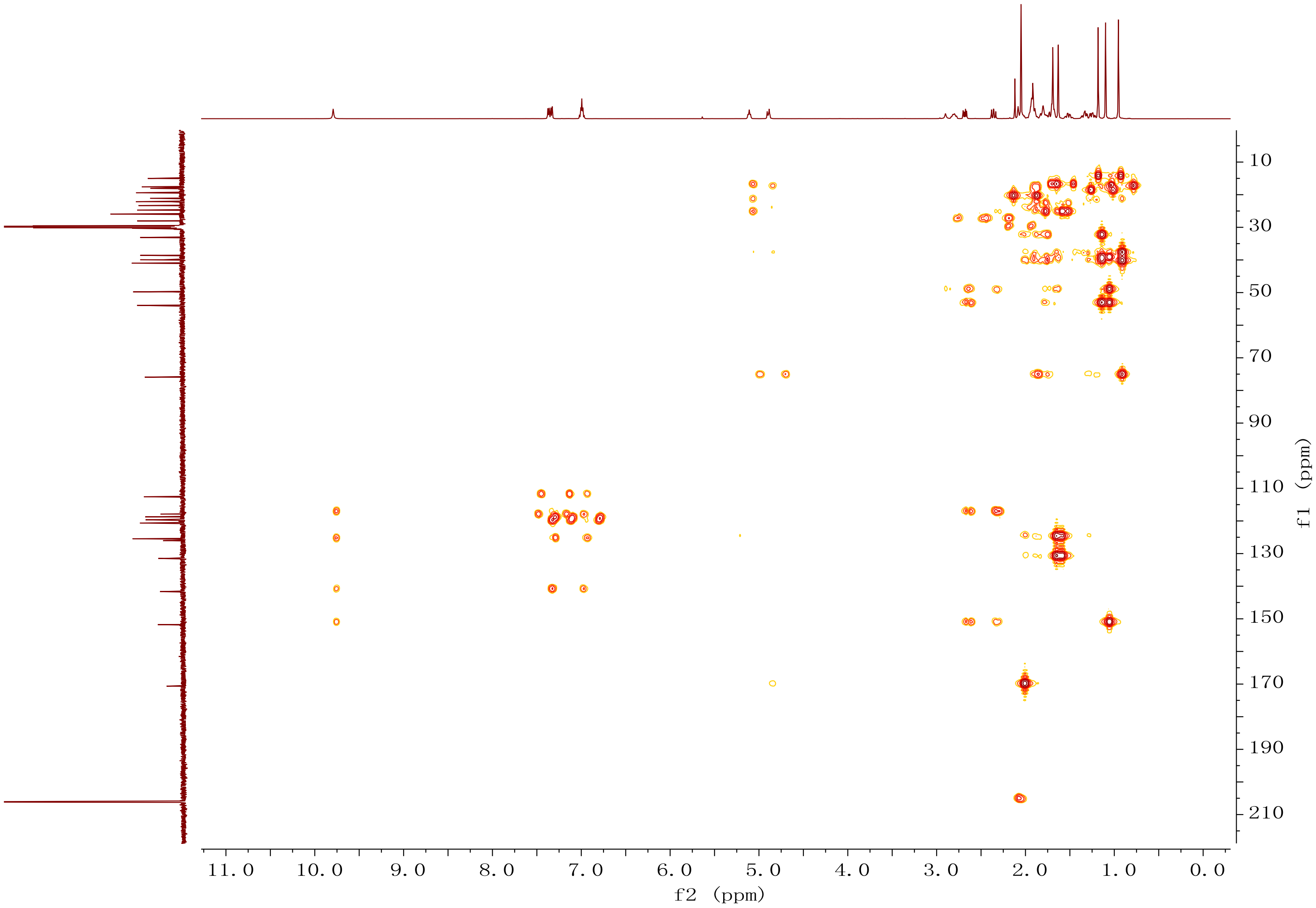

S17. NOESY spectrum of compound **3**.

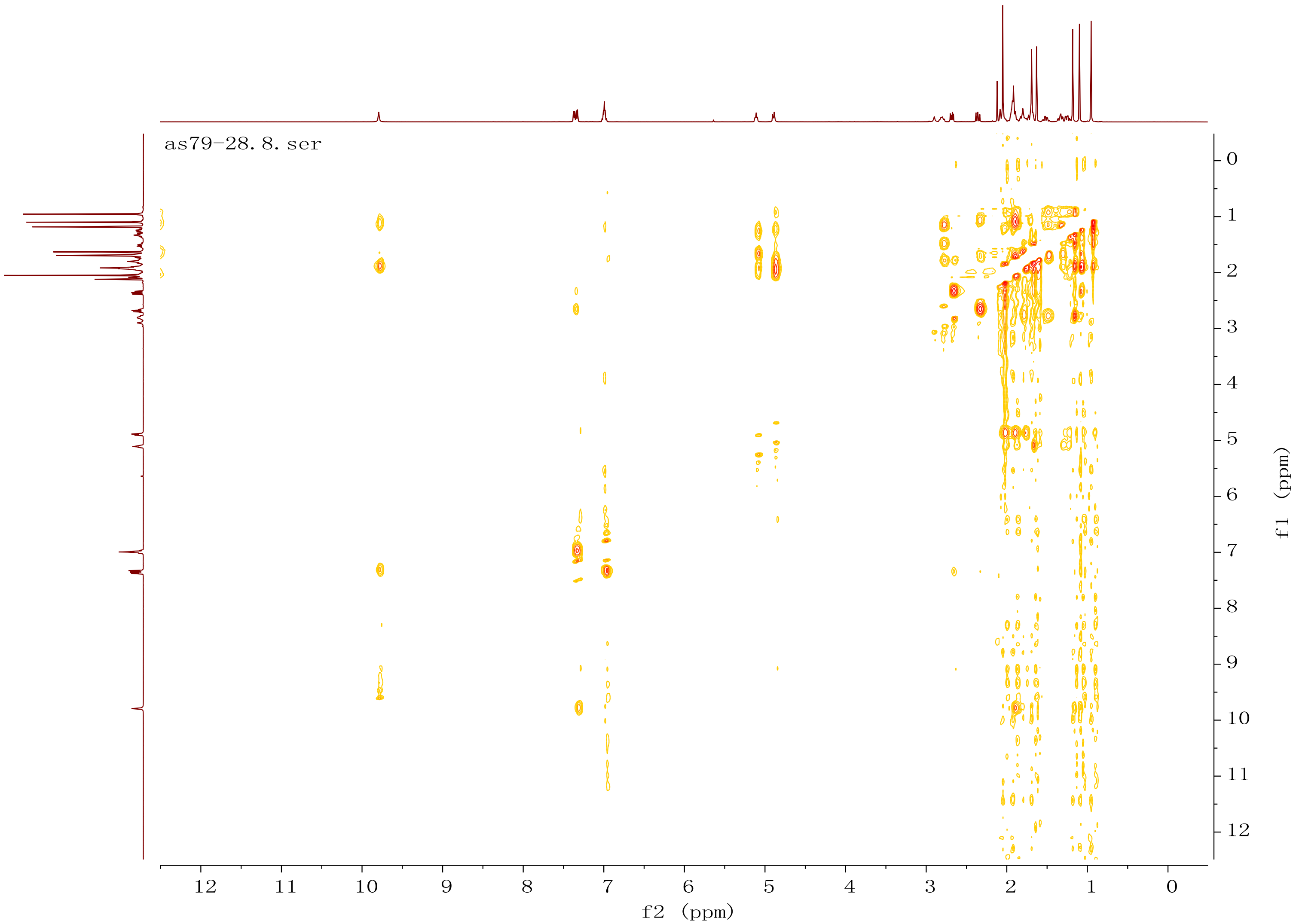

S81. Crystal packing of compound **3** at 293(2) K.

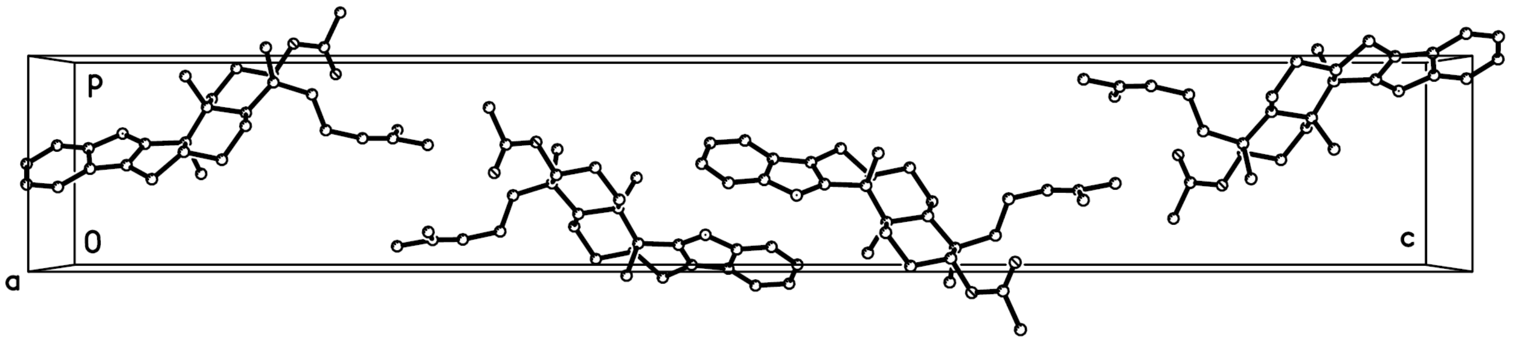

Supplement: Supplementary file 1 [file marinedrugs-15-00137-s001.pdf]
